# Effects of HIV-1 infection on malaria parasitemia in milo sub-location, western Kenya

**DOI:** 10.1186/s13104-015-1270-1

**Published:** 2015-07-15

**Authors:** Erick Kipkoech Rutto, Joshua Nyagol, Julius Oyugi, Samson Ndege, Noel Onyango, Andrew Obala, Chrispinus J Simiyu, Gye Boor, Winfrida Chelangat Cheriro, Barasa Otsyula, Ben Estambale

**Affiliations:** Institute of Tropical and Infectious Diseases, University of Nairobi, Nairobi, Kenya; Department of Human Pathology, College of Health Sciences, University of Nairobi, Nairobi, Kenya; Department of of Clinical Medicine and Therapeutics, College of Health Sciences, University of Nairobi, Nairobi, Kenya; Department of Medical Microbiology College of Health Sciences, University of Nairobi, Nairobi, Kenya; Department of Medical Microbiology and Parasitology, Moi University, Eldoret, Kenya; Department of Epidemiology and Nutrition, Moi University, Eldoret, Kenya; Department of Surgery and Anaethesiology, Moi University, Eldoret, Kenya; Moi Teaching and Referral Hospital (MTRH), Eldoret, Kenya

**Keywords:** HIV-1, Malaria, Malaria density

## Abstract

**Background:**

Malaria and HIV infections are both highly prevalent in sub-Saharan Africa, with HIV-infected patients being at higher risk of acquiring malaria. HIV-1 infection is known to impair the immune response and may increase the incidence of clinical malaria. However, a positive association between HIV-1 and malaria parasitaemia is still evolving. Equally, the effect of malaria on HIV-1 disease stage has not been well established, but when fever and parasitemia are high, malaria may be associated with transient increases in HIV-1 viral load, and progression of HIV-1 asymptomatic disease phase to AIDS.

**Objective:**

To determine the effects of HIV-1 infection on malaria parasitaemia among consented residents of Milo sub-location, Bungoma County in western Kenya.

**Study design:**

Census study evaluating malaria parasitaemia in asymptomatic individuals with unknown HIV-1 status.

**Methods:**

After ethical approvals from both Moi University and MTRH research ethics committees, data of 3,258 participants were retrieved from both Webuye health demographic surveillance system (WHDSS), and Academic Model Providing Access to Healthcare (AMPATH) in the year 2010. The current study was identifying only un-diagnosed HIV-1 individuals at the time the primary data was collected. The data was then analysed for significant statistical association for malaria parasitemia and HIV-1 infection, using SPSS version 19. Demographic characteristics such as age and sex were summarized as means and percentages, while relationship between malaria parasitaemia and HIV-1 (serostatus) was analyzed using Chi square.

**Results:**

Age distribution for the 3,258 individuals ranged between 2 and 94 years, with a mean age of 26 years old. Females constituted 54.3%, while males were 45.8%. In terms of age distribution, 2–4 years old formed 15.1% of the study population, 5–9 years old were 8.8%, 10–14 years old were 8.6% while 15 years old and above were 67.5%. Of the 3,258 individuals whose data was eligible for analysis, 1.4% was newly diagnosed HIV-1 positive. Our findings showed a higher prevalence of malaria in children aged 2–10 years (73.4%), against the one reported in children in lake Victoria endemic region by the Kenya malaria indicator survey in the year 2010 (38.1%). There was no significant associations between the prevalence of asymptomatic malaria and HIV-1 status (p = 0.327). However, HIV-1/malaria co-infected individuals showed elevated mean malaria parasite density, compared to HIV-1 negative individuals, p = 0.002.

**Conclusion:**

HIV-1 status was not found to have effect on malaria infection, but the mean malaria parsite density was significantly higher in HIV-1 positive than the HIV-1 negative population.

**Electronic supplementary material:**

The online version of this article (doi:10.1186/s13104-015-1270-1) contains supplementary material, which is available to authorized users.

## Background

The clinical burden of malaria and HIV/AIDS in sub-Saharan Africa is well-described, but the dynamics of the interaction between the two diseases remains poorly understood. Malaria and HIV infections are both highly prevalent in sub-Saharan Africa, with HIV-infected patients being at higher risk of acquiring malaria [1]. Conversely, HIV-1 infection is known to impair the immune response, and increase the incidence of clinical malaria. However, a positive association between HIV-1 and malaria parasitaemia has not been fully explored. Equally, the effect of malaria on HIV-1 disease stage has not been well established, but when fever and parasitemia are high, malaria may be associated with transient increase in HIV-1 viral load and progression of HIV-1 asymptomatic disease phase to AIDS [2, 3].

In Kenya, malaria remains one of the greatest public health problems, contributing to 30% of all the outpatient consultations, and 19% of hospital admissions [4]. In addition, HIV-1 prevalence among 15–49 years old as per the Kenya Demographic and Health Survey of 2008–2009 was 6.3%, with reported 44 new infections every day [4, 5]. By the year 2002 HIV-1 and malaria were the leading causes of mortality in the African region [6]. In addition, HIV-1 and malaria have currently been documented to be very common in sub-Saharan Africa, with hypothesized potential of interactions. Malaria has been implicated in the increase of HIV-1 viral load and, as a result, a decrease in CD4^+^ T cells, high rates of HIV-1 transmission and rapid disease progression to AIDS. On the other hand, immunosuppressive effects due to HIV-1 infection has been associated with increased episodes of clinical malaria, and increased fatality rates in endemic regions [7–9].

A number of studies have shown some correlations in interactions between HIV-1 and malaria infections on patients’ outcome [10–12]. However, there is still emerging evidence of malaria and HIV-1 co-infection dynamics and relationships. Combined anti-retroviral (cARV) and anti-malarial prophylactic drug interactions are also known to pose a serious threat to their efficacy, and could lead to synergistic toxicities, given that majority of cART and anti-malarial drugs are metabolized by the CYP450 system, creating a chance of drug–drug interaction upon co-administration [13].

These multiple interactions have serious implications on service delivery and are of public health concern in the management of HIV-1 and malaria epidemics. Therefore, in HIV-1 infected individuals, a malaria case definition based on fever alone, which is a regular management tool for clinically diagnosing malaria in resource limited areas may be misleading. Furthermore, in advanced HIV-1 disease, anaemia is a common hematological presentation, in which malaria is thought to have a contributory role. HIV-1 co-infection has been implicated in increased risk of malaria associated anemia and poor recovery, than in HIV-1 negative patients [11].

The current study, therefore, sought to investigate the effects of untreated HIV-1 infection on malaria parasitaemia among residents of Milo sub-location, Webuye district in Bungoma County in western Kenya. Western Kenya has an HIV-1 prevalence of 6.6% among men and women aged between 15 and 49 years, a 0.3% higher than that of national mean for Kenya [5]. It is also a region with malaria endemicity, making it a good model for the study of association between HIV-1 infection and its affects on malaria parasitaemia.

## Methods

### Study population

Milo sub-location, in which the project was conducted, is one of the six sub-locations within Webuye Health Demographic Surveillance System (WHDSS) in Bungoma County Western Kenya. It is a transitional zone between areas of stable and unstable malaria transmissions, with a high prevalence of HIV-1. This provided a potential ground to investigate the interactions of these two infectious agents.

After ethical approvals from both Moi University and MTRH research ethics committees, data from 3,258 consented participants as well as children whose parents or guardians gave assent, and who were involved in the malaria and HIV-1 survey were retrieved. These were adults and children above 2 years old, and had lived for more than 4 months within Milo sub-location. From the retrieved data, the current study comprised of individuals with asymptomatic malaria, and were not aware of their HIV-1 status.

After authorization to use data was granted by both AMPATH and WHDSS, demographic details and information on malaria were retrieved from Webuye Health Demographic Surveillance System (WHDSS) data base, while data on HIV-1 was accessed from the records of Home based counseling and testing for HIV-1, that was conducted by AMPATH in the year 2010. Participants’ confidentiality was maintained by ensuring that names or any other special identifiable characteristics of an individual participant did not appear in the study.

### Malaria parasitaemia

For the purpose of WHDSS data base, a finger-prick blood drop was taken from individual participants to make a thick smear and then stained with 10% Giemsa. The slides were examined by two independent trained technologists, and results recorded as positive or negative for malaria. Where discordance arose, an independent qualified assessor re-examined the discordant blood slide(s) readings, and the findings were then adopted. Malaria positive smears were used to determine the malaria parasite density (MPD) for each participant, by counting parasites in each thick blood smears. In every total 200 white blood cells (WBC) assessed, malaria parasites were then calculated for each positive smear, and reported per micro-litre (µl) of blood using the following standard formula as per the WHO [12].

$${\text{Parasites per }}\upmu {\text{l of blood}} = \frac{{{\text{No}}.\,{\text{malaria}}\,{\text{parasites}}\,{\text{counted}}\, \times \,8,000\,{\text{WBC}}}}{{200{\text{ WBC}}}}.$$

### HIV sero-status

For the purpose of AMPATH database, data on HIV-1 sero-status was collected concurrently with WHDSS malaria parasitaemia surveillance. Informed consent was obtained for HIV-1 testing for every individual in the community in which house to house visits were conducted, followed with a finger prick to obtain drops of blood for HIV testing. For the minors, assent was obtained from the parents or their guardians. Three standard rapid tests (Determine^®^ HIV-1/2; Inverness Medical Japan, SDBioline^®^ HIV 1/2 3.0; Standard Diagnostics Inc. and Unigold^®^ Trinity biotech PLC, Ireland) were used to determine the HIV status according to manufacturers’ specifications. First, Determine^®^ HIV1/2 was used and results recorded. For negative individuals, no further tests were done. For positive reactions, the results were verified using SD Bioline^®^ HIV1/2, in which case a positive test confirmed HIV sero-positivity. If the two tests were discordant, a third rapid test was done using Unigold^®^ to reconcile the results. In addition, fourth generation ELISA (Vironostika, France) was done for discordant couples, and every 20th specimen for quality assurance and quality control. Results were communicated to the participants and counseling done accordingly. Those who tested positive were thereafter enrolled to the nearby AMPATH clinic for further counseling, evaluation and care.

### Data analysis

Data was imported to SPSS from excel spread sheet, and analyzed using SPSS version 19 (SPSS, Chicago IL, USA). Demographic characteristics such as age and sex were summarized into means and percentages. Association between HIV-1 and malaria prevalence was subjected to Chi square analysis for significant statistical correlation, while adjusting for age. Values of p < 0.05 were considered statistically significant.

## Results

### Demographic details

Data for 3,258 consented participants was eligible for analysis, which constituted 97% of the total population in the records. Females constituted 54.3%, while males were 45.8%. In terms of age distribution, 2–4 years old formed 15.1%, 5–9 years old were 8.8%, 10–14 years old were 8.6% while 15 years old and above were 67.5% of the participants. The prevalence of newly diagnosed HIV-1 positive individuals was 1.4% (46 out of the 3,258 participants), with the highest HIV-1 positive individuals being between 15 and 49 years. Out of the HIV-1 positive category, females were 71.7%, while males were 28.3%.

### Prevalence of malaria parasitemia (*Plasmodium falciparum*) and HIV-1 co-infection

From the consented participants, 64.3% tested positive, while 35.7% were negative for malaria parasite. For the HIV-1 negative cluster 35.6% were positive, while 64.4% were negative for malaria parasite. In the HIV-1 positive cluster, 58.7% were positive, while 41.3% were negative for malaria parasite. However, no significant statistical relationship between malaria parasitemia and HIV-1 co-infection was found (*P* = 0.327) (Additional file 1: Table S1).

### Malaria parasite density

Mean malaria parasite density was compared between the HIV-1 positive and HIV-1 negative individuals, and was found to be 2,008.9/µl, SD 7,602.3 and 1,043.7/µl, SD 3,029.5 in HIV-1 positive and HIV-1 negative categories, respectively (*P* = 0.002). The medians for the parasite densities were 440 and 480 for HIV-1 positive and HIV-1 negative categories, respectively (Additional file 1: Table S1).

## Discussion

Both malaria and HIV-1 infections are still highly prevalent in sub-Saharan Africa, with HIV-1 infected patients being at higher risk of acquiring malaria [13]. In addition, HIV-1 infection has been associated with an increase in malaria parasite density, delayed parasite clearance, a higher incidence of clinical and severe malaria, as well as increased death rate. Other studies have also suggested that there is an increase in plasma HIV-1 RNA levels observed during an episode of clinical malaria, which involves a complex interplay of several factors. However, despite the growing body of evidence on the pathophysiological and epidemiological interactions between HIV-1 and malaria in sub-Saharan Africa, the dynamics of the interaction between the two diseases remain poorly understood [14–17].

The present study showed that, in a region considered to be hyperendemic for malaria, even though it is a transitional zone between hyperendemic and meso endemic malaria zones, the overall malaria prevalence was 64.3% [18, 19]. For the age group 2–10 years, malaria prevalence was 73.4%, which was higher than 38.1% reported in children in lake Victoria endemic region by the Kenya malaria indicator survey in the year 2010. This disparity could be attributed to a wider region of coverage by the KMIS in 2010, resulting into a lower mean, as compared to the result from our study. However, data for our study was obtained from a survey study conducted during malaria peak season of October to November 2010. Similarly, the Kenya malaria indicator survey data resulted from a different peak malaria season of June–August 2010 [18]. A closer comparison to our findings was from a study of population based survey of *Plasmodium falciparum* in western Kenya highlands carried out during the months of July–December in the year 2002, which found a prevalence of malaria among children aged between 2 and 4 years to be 62.8% [19].

Although the percentage positive for malaria parasitaemia in the HIV-1 positive category in our study was higher compared to the HIV-1 negative cluster, infection with the virus did not seem to have any significant effect on the prevalence of malaria in the assessed individuals. [20–22]. However, the mean malaria parasite density in HIV-1 infection was found to have impact on malaria parasitaemia, compared with HIV-1 negative group, a factor likely associated with immunosuppressive effects of HIV-1 [22, 23].

Until now, there is no consensus regarding the effect of HIV-1 on malaria. Some earlier studies found no effect of HIV-1 on the susceptibility and severity of malaria, but recent investigations suggest that HIV-1-infected individuals with lower CD4 cell counts may be more likely to present with symptomatic malaria, and malaria may enhance HIV-1 vertical transmission [24]. The above phenomena has, however, been contradicted by some studies showing that HIV-1 replication is increased 10 to 100-fold in peripheral blood mononuclear cells exposed to soluble malaria antigens and [25].

In addition, it has been shown that in regions where HIV and malaria parasites are both highly endemic, acute bouts of malaria drive higher plasma HIV RNA levels [26]. Decline in the CD4^+^ T cell count has also been reported to be accelerated by clinically apparent bouts of *Plasmodium falciparum* malaria [27]. Although clinical data have not yet been demonstrated, it is quite possible that subclinical parasitemia as it was for our study may also contribute to this phenomenon of detection of HIV in asymptomatic malaria positive individuals.

There are, though, some limitations to this study. First, this was a surveillance study that could not analyze in details the complex interplay of the effects of HIV on malaria by evaluating levels of CD4^+^/CD8^+^ T cells ratio and HIV disease stage. In addition, the study assessed asymptomatic malaria individuals who were not on any medication for either malaria or HIV management. This is in light of the fact that several studies have shown that combination antiretroviral therapy has great potential to reduce HIV-related malaria. Cotrimoxazole prophylaxis, recommended for adults and children living with HIV in Africa, for instance, has been reported to be effective in reducing clinical malaria, independent of baseline CD4 [28, 29].

## Conclusion

Our findings indicate that HIV infection did not show any influence on malaria parasitemia. However, the mean malaria parasite density was significantly higher in HIV positive group than the HIV negative population, a challenging finding that should necessitate further studies on the link between HIV infection and malaria parasite density levels.

## Additional file

Additional file 1:
**Table S1.** Expressions age, sex, malaria parasitaemia and density against HIV status.

## Abbreviations

AIDS: acquired immunodeficiency syndrome
AMPATH: Academic Model Providing Access to Healthcare
ELISA: enzyme linked immunosorbent assay
HIV: human immunodeficiency virus
KMIS: Kenya malaria indicator survey
MTRH: Moi Teaching and Referral Hospital
MPD: Malaria parasite density
SPSS: statistical program for social sciences
WHDSS: Webuye health demographic surveillance system
