# Supplementary material for: Effects of HIV-1 infection on malaria parasitemia in milo sub-location, western Kenya
Source: BMC Res Notes. 2015 Jul 15;8:303. doi: 10.1186/s13104-015-1270-1 (PMC4501056; doi:10.1186/s13104-015-1270-1)
Supplement: Additional file 1: — Table S1. Expressions age, sex, malaria parasitaemia and density against HIV status. [file 13104_2015_1270_MOESM1_ESM.doc]

|  | **HIV STATUS** | |
| --- | --- | --- |
|  | **POSITIVE (+); n=46** | **NEGATIVE (-); n= 3212** |
| **Age Distribution** |  |  |
| 2-14 yrs | 10.9% | 32.7% |
| 15-49 yrs | 71.7% | 53.0% |
| ≥50 yrs | 17.4% | 14.3% |
| **Sex** |  |  |
| Male | 28.3% | 46.1% |
| Female | 71.7% | 53.9% |
| **Malaria Parasite** |  |  |
| Positive (+) | 58.7% | 35.6% |
| Negative (-) | 41.3% | 64.4% |
| **Mean malaria parasite density and SD** | 2008.9; SD= 7602.3 | 1043.7; SD= 3029.5 |

Table S1. Expressions age, sex, malaria parasitaemia and density against HIV status
